# Gender differences in clinical presentations and sensory profiles in patients with fibromyalgia: implications of peripheral and central mechanisms

**DOI:** 10.1097/PR9.0000000000001229

**Published:** 2025-01-13

**Authors:** Min Liu, Stephany Harris, Anna P. Andreou, Xuenong Bo, Adnan Al-Kaisy

**Affiliations:** a Pain Management and Neuromodulation Centre, Guy's and St. Thomas' Hospital, London, United Kingdom; b Centre for Neuroscience, Surgery and Trauma, Queen Mary University of London, London, United Kingdom

**Keywords:** Fibromyalgia, Sensory profiles, QST, quantitative sensory testing, Gender

## Abstract

**Introduction:**

Fibromyalgia has a high female predominance and research work has been focussing mainly on women.

**Objectives:**

We aimed to answer (1) gender differences in pain scores and quality of life, (2) any gender-specific subgroups defined by quantitative sensory testing (QST), and (3) correlations of QST parameters with pain intensity and questionnaire scores.

**Methods:**

We evaluated clinical presentations and QST profiles from 38 male and 38 age-matched female patients.

**Results:**

Women reported significantly higher scores in average daily pain, daily sleep interference score, average weekly pain, weekly sleep interference score, and revised fibromyalgia impact questionnaire (rFIQ). Based on LOGA classification, L0G2, mechanical allodynia or hyperalgesia without abnormal sensory loss, was the most common QST subtype which accounted for 28.9% of men and 26.3% of women. Approximately 34.2% of men and 26.3% of women displayed loss of function of small fibres with an increased cold or warm detection threshold. Cold detection threshold was negatively correlated with pain intensity and functional impairment, suggesting a peripheral mechanism. Central sensitization, defined as allodynia and hyperalgesia to thermal or mechanical stimuli, was found in two-thirds of male and female patients. Mechanical pain sensitivity was positively correlated with the severity of pain and associated symptoms in women, but not men.

**Conclusion:**

There was a marked gender difference in reported pain and quality of life. We have confirmed that central sensitization is a major mechanism for women. Our data suggested an important role of small fibre pathology in both men and women.

## Abstract 2

Gender plays an important role in clinical presentations and sensory profiles in patients with fibromyalgia. Quantitative sensory testing reveals underlying peripheral and central mechanisms.

## 1. Introduction

Fibromyalgia is a chronic condition characterized by widespread musculoskeletal pain that affects 2% to 8% of the world population.^8^ The incidence is markedly higher in women with a female-to-male ratio of 9:1. However, epidemiology studies have revealed that men could make up to 40% of overall patients. A population survey of over 4000 patients using fibromyalgia criteria 2016 has found a female-to-male ratio of 6:4.^47^ The incidence of fibromyalgia in the general population was found to be 3.98% and 2.40% in women and men in a systemic review.^18^ The prevalence could be as high as 5% to 15% in the elderly male population based on European male ageing study.^26^ In clinical practice, the diagnosis of male patients could be underestimated due to gender bias by physicians and report bias by patients.^47^ The research on male patients has been hampered by the seemingly lower incidence.

It is well established that women are more likely to report a variety of pains, more severe pain, more frequent pain, and pain of longer duration.^44^ There are gender differences in pain biology. For example, female patients have lower pain thresholds in thermal and mechanical pain.^36^ In addition, sociocultural and psychological variables have been identified as contributing factors to the gender difference.^5^ The gender has been found to influence pain severity and quality of life in patients with fibromyalgia.^9^ For example, female patients with fibromyalgia reported higher scores in pain and symptom severity than male patients.^7,20,31,47,50^ However, conflict results have shown that men with fibromyalgia reported severer symptoms.^2,6,37^ It is important to clarify the gender differences in clinical presentations.

Central sensitization is considered a major mechanism in fibromyalgia. It is defined as an increased responsiveness in the central nervous system (CNS) to either normal or subthreshold afferent input. Gracely et al.^16^ have found cortical or subcortical augmentation of pain processing based on a functional magnetic resonance imaging study. When temporal summation of pain (wind-up) was assessed using a series of repetitive thermal stimulation, the perceived magnitude of the sensory response was greater in individuals with fibromyalgia.^34,42^ Studies have found a positive correlation between central sensitization and pain intensity.^19,35^ In addition to the central mechanism, several lines of evidence indicate peripheral mechanisms including small fibre pathology,^17,32,43^ dysregulation of the immune process,^23,30^ and autoantibodies.^15^ Many silent nociceptors from patients with fibromyalgia showed hyperexcitability on microneurographic recording.^39^ Sluka and Clauw^40^ have proposed that fibromyalgia is a continuum with one end as a purely peripherally driven painful condition and the other as predominantly central enhancement in pain processing. The German Research Network on Neuropathic Pain (DFNS) has established a quantitative sensory testing (QST) protocol to examine abnormal sensory loss or gain.^36^ Age-matched and gender-matched absolute and relative QST reference values have been established for the cheek, dorsum of hand, and foot.^27^ Different patterns of sensory abnormalities likely reflect altered functions in somatosensory processing and suggest distinct underlying pain mechanisms.^10,28^ A DFNS study demonstrated several QST profiles in patients with neuropathic pain including normal, sensory losses, sensory gains, or both.^28^ In this study, we analysed pain scores, quality of life, and QST profiles to verify gender differences and possible mechanistic implications.

## 2. Materials and methods

### 2.1. Study cohort and design

This study was a single-centre retrospective analysis of adult patients with fibromyalgia from 2019 to 2022. A clinical diagnosis of fibromyalgia was based on the revised American College of Rheumatology (ACR) 2016 diagnostic criteria.^46^ Exclusion criteria were as follows: autoimmune disease, neurological disorders, psychiatric disorders (excluding anxiety, depression, and post-traumatic stress disorder), and pregnancy. Previous studies have revealed a 1-point difference between male and female patients in pain severity.^20^ Considering an effect size of 1, a confidence level of 95%, a desired power of 80%, and an SD of pain severity of 1.5 from an epidemiological study,^47^ the estimated sample size was 35. We have collected data from 38 male and 178 female patients. A major limitation of previous clinical studies is the small number of men compared with women.^3,4,20,24^ In this study, we included data from 38 male patients and an equal number of age-matched female patients. Other available data will be presented in our manuscript on the efficacy and predictors of systemic lidocaine infusion in patients with fibromyalgia.

### 2.2. Questionnaires, screening blood tests, and clinical investigations

The Widespread Pain Index (WPI, 0–19) and symptom severity score (SSS, 0–12) were based on patient-reported symptoms. The severity of pain was rated for the daily worst, least, and average pain using the 11-point numerical rating scale (NRS, 0 = no pain, 10 = worst possible pain). The daily sleep interference score (DSIS) was measured on a similar rating scale (0 = no interference, 10 = complete interference). The weekly pain and sleep interference scores (AWP and WSIS) were the means of average daily pain (ADP) and DSIS for 7 days. The revised fibromyalgia impact questionnaire (rFIQ) contains 21 items covering 3 domains: functional impairment, overall impact, and symptom severity. All questions were graded on the 11-point numerical rating scale, and the total score of rFIQ is 100.

### 2.3. Quantitative sensory testing

The QST was performed on the dorsum of the right foot following a standardized protocol.^36^ The following parameters were assessed: cold and warm detection thresholds (CDT and WDT), the ability to detect temperature changes thermal sensory limen (TSL), cold and heat pain thresholds (CPT and HPT), mechanical detection and pain thresholds (MDT and MPT), mechanical pain sensitivity (MPS), dynamic mechanical allodynia (DMA), wind-up ratio (WUR), and vibration detection. Vibration was recorded as intact or absent. Pressure pain threshold (PPT) was not tested consistently; therefore, it was excluded from this study. The combination of sensory gains and losses was analysed using the LOGA classification.^28^ Loss of detection for thermal stimuli (CDT or WDT) was coded as L1, for mechanical stimuli (MDT or VDT) as L2, and loss of both as L3. Gain of function for thermal stimuli (CPT or HPT) was classified as G1, for mechanical stimuli (MPT, MPS, DMA, and WUR) as G2, and gain for both as G3. L0 represented no loss for thermal or mechanical detection and G0 meant no thermal or mechanical allodynia or hyperalgesia. Loss of function of small fibres was indicated by increased CDT and/or WDT, and loss of function of large fibres by increased MDT or loss of vibration detection. Central sensitization was defined as thermal hyperalgesia (G1), mechanical allodynia or hyperalgesia (G2), or both (G3).

### 2.4. Statistical analysis

IBM SPSS statistics version 25 was used for statistical analysis. Quantitative sensory testing parameters were compared with control values corresponding to age, and gender, and with the dorsum of the foot as a reference site.^27^ Using logarithmic transformation of the raw data, Z-scores were calculated as follows: Z-score = (value of the subject − mean value of controls)/standard deviation. The 95% confidence interval of healthy controls is between −1.96 and +1.96. Abnormal values were defined as Z-scores outside 95% confidence interval (< −1.96 = abnormal loss; >1.96 = abnormal gain). Descriptive statistics was applied to summarize the variables. The continuous variables were reported as mean ± SD (standard deviation), and the discrete variables were expressed as the number of observations and frequency. Interval variables including pain, sleep interference, and rFIQ scores were expressed as mean ± SD, and compared using Student's *t* test for 2 groups. The categorical variables were examined using chi-square test with or without Yates correction. Linear correlation of QST parameters, pain score, and questionnaire results were assessed by the Kendall tau-b correlation coefficient. *P* < 0.05 was considered statistically significant.

## 3. Results

### 3.1. Male and female patients had similar widespread pain index, symptom severity score, and incidence of depression

Table 1 presents the demographic data of 38 male and 38 age-matched female patients. There was no statistical significance in BMI, although female patients had a slightly higher mean value. The percentage of obesity (BMI ≥ 30) was 26.3% (range: 19.1–36.3) in male patients and 34.2% (range: 18.5–41) in female patients. The WPI and SSS were similar in male and female patients. The average onset age of widespread pain was marginally earlier in male than in female patients (34.1 ± 10.1 vs 37.8 ± 10.7) without statistical significance. We have found that 57.9% of male patients were diagnosed with depression, in comparison with 65.8% of female patients (chi-square test, *P* = 0.478).

**Table 1:** Clinical and diagnostic test data.

	Male	Female	t value	*P*
Age (y)	49.7 ± 10.0	48.7 ± 11.7	0.401	0.689
BMI (kg/m^2^)	28.0 ± 4.2	29.7 ± 6.1	1.126	0.266
WPI	14.0 ± 3.6	14.6 ± 4.2	0.725	0.471
SSS	8.8 ± 2.4	9.4 ± 1.9	1.195	0.236
Pain duration (y)	14.6 ± 8.1	15.0 ± 11.6	0.183	0.855
Onset of pain (y)	34.1 ± 10.1	37.8 ± 10.7	0.154	0.878

The demographic data including age, BMI (body mass index), duration of pain, and onset of generalised pain were expressed as mean ± SD (n = 38). The WPI (widespread pain index) and SSS (symptom severity score), the diagnostic criteria of fibromyalgia, were expressed as mean ± SD. The Student's *t* test was used to examine possible statistical significance.

### 3.2. Male patients reported significantly lower scores in pain, sleep interference, and revised fibromyalgia impact questionnaire

**Figure 1.**
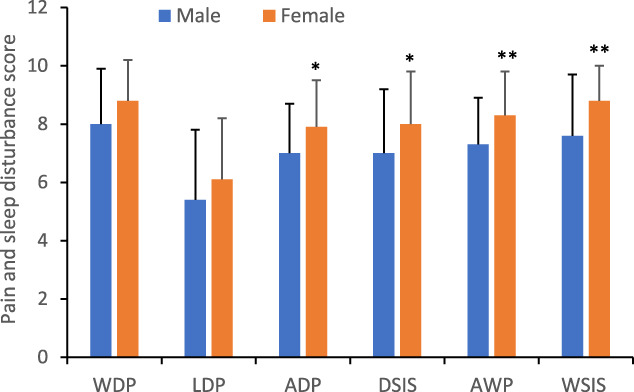
Scores of pain and sleep interference in male and female patients. The data were expressed as mean ± SD. *P < 0.05; **P < 0.01. ADP, average daily pain; AWP, average weekly pain; DSIS, Daily Sleep Interference Score; LDP, daily least pain; WDP, daily worst pain; WSIS, weekly sleep interference score.

**Figure 2.**
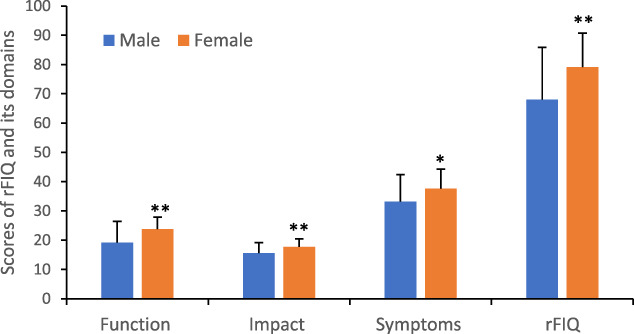
A comparison of quality of life in male and female patients. rFIQ: revised fibromyalgia impact questionnaire; function, impact, and symptoms are the 3 domains in the rFIQ. The data were expressed as mean ± SD. *P < 0.05; **P < 0.01.

Both male and female patients reported moderate-to-severe daily pain (Fig. 1). The scores of least and worst daily pain (LDP and WDP) were 5.4 ± 2.4 to 8.0 ± 1.9 in male and 6.1 ± 2.1 to 8.8 ± 1.4 in female patients. The average daily pain (ADP) was significantly higher in female patients. The daily sleep interference score (DSIS) reported by female was 1 point higher than that reported by male patients. Similarly, the average weekly pain (AWP) and weekly sleep interference score (WSIS) were significantly higher in female patients. The rFIQ contain 3 components: function, impact, and symptoms (Fig. 2). The female patients had significantly higher scores in all domains and the total score. The latter was 68.0 ± 17.8 in male patients and 79.1 ± 11.6 in female patients (Student's *t* test, *P* = 0.002).

### 3.3. Loss of function of small and large fibres was more prevalent in men, and gain of function was the major phenotype in both men and women

**Figure 3.**
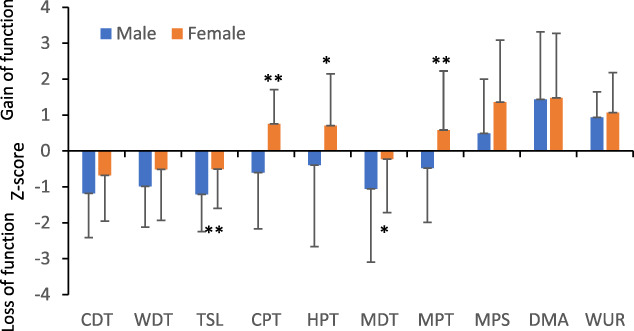
A comparison of QST parameters in male and female patients. The data were expressed as mean ± SD. *P < 0.05; **P < 0.01. CDT, cold detection threshold; CPT, cold pain threshold; DMA, dynamic mechanical allodynia; HPT, heat pain threshold; MDT, mechanical detection threshold; MPS, mechanical pain sensitivity; MPT, mechanical pain threshold; QST, quantitative sensory testing; TSL, thermal sensory limen; WDT, warm detection threshold; WUR, wind-up ration.

**Figure 4.**
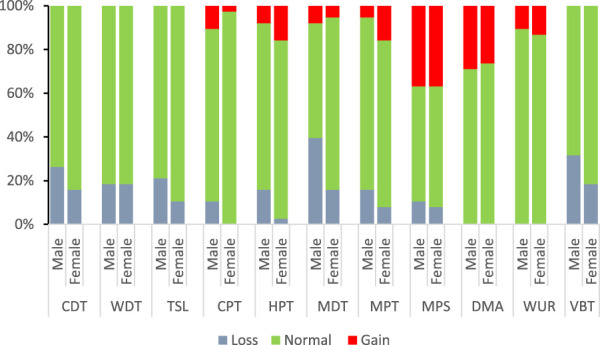
The percentage of patients who had normal, loss, and gain of function in QST parameters. Z-scores outside 95% confidence interval are defined as abnormal (< −1.96 = abnormal loss; > +1.96 = abnormal gain). QST, quantitative sensory testing.

Figure 3 shows the mean values (±SD) of QST parameters. In comparison, male patients had a significant loss of function in TSL and MDT, while female patients had a significant gain of function in CPT, HPT, and MPT. Figure 4 shows the percentage of normal, loss and gain of function in QST measurements in male and female patients. Loss of function in CDT was seen in 26.3% and 15.8% of male and female patients (chi-square test, *P* = 0.26). A similar pattern was seen in TSL without statistical significance. The percentage of loss of function in MDT and vibration was higher in male (39.4% and 31.6%) than in female patients (15.8% and 18.4%), but only loss of function in MDT reached statistical significance (*P* = 0.021). The highest percentage of gain of function was seen in MPS in both male and female patients (36.8%). That was followed by DMA (28.9% and 26.3%), WUR (10.5% and 13.2%), and MPT (5.3% and 15.8%) in male and female patients, respectively. The cold and heat hyperalgesia, manifested as a gain of function in CPT and HPT, was seen in 10.5% and 7.9% of male, and in 2.6% and 15.8% of female patients.

Table 2 presents the subtypes of abnormal sensory loss and gain according to LOGA classification.^28^ The most prevalent LOGA subtype was mechanical hyperalgesia without sensory loss (L0G2), found in 28.9% and 26.3% of male and female patients, respectively. The loss of function in small (CDT and WDT) and large (MDT and vibration) fibres was more prevalent in male than female patients. Loss of function of small fibre was found in 34.2% of male (L1: 15.8% and L3: 18.4%) and 26.3% of female patients (L1: 21% and L3: 5.3%). Loss of function of large fibres was seen in 39.4% of male (L2: 21% and L3: 18.4%) and 21.1% of female patients (L2: 15.8% and L3: 5.3%). Abnormal sensory loss (L1 + L2 + L3) was more common in male compared with that of female patients (55.3% vs 42.1%), but failed to reach statistical significance (chi-square test, *P* = 0.242). Mechanical hyperalgesia was far more common than thermal hyperalgesia in both male and female patients (Yates-corrected chi-square test, *P* < 0.001). Abnormal sensory gain (G1 + G2 + G3), defined as central sensitization, was found in 63.2% of male and 68.4% of female patients. Notably, a normal QST examination (L0G0) was seen in 15.8% of female but only in 7.9% of male patients.

**Table 2:** Different combinations of gain and loss of function in male and female patients.

	Male	Female	All
L0 (no loss)	17 (44.7%)	22 (57.9%)	39 (51.3%)
L1 (thermal)	6 (15.8%)	8 (21.0%)	14 (18.4%)
L2 (mechanical)	8 (21.0%)	6 (15.8%)	14 (18.4%)
L3 (thermal + Mechanical)	7 (18.4%)	2 (5.3%)	9 (11.8%)
All	38 (100%)	38 (100%)	76 (100%)
G0 (no gain)	14 (36.8%)	12 (31.6%)	26 (34.2%)
G1 (thermal)	1 (2.3%)	4 (10.5%)	5 (6.6%)
G2 (mechanical)	19 (50%)	19 (50%)	38 (50.0%)
G3 (thermal and mechanical)	4 (10.5%)	3 (7.9%)	7 (9.2%)
All	38 (100%)	38 (100%)	76 (100%)

L0: no loss of thermal or mechanical detection; L1: only loss of thermal detection defined as an increase in CDT or WDT (loss of small-fibre function); L2: only loss of mechanical detection defined as an increase in MDT or VDT (loss of large-fibre function); L3: loss of both thermal and mechanical detection; G0: no gain (no thermal or mechanical allodynia or hyperalgesia); G1: only thermal hyperalgesia defined as a gain of function in HPT or CPT; G2: mechanical allodynia or hyperalgesia defined as a gain of function in MPS, MPT, DMA, and WUR; G3: thermal and mechanical allodynia and hyperalgesia. The data were expressed as the number and percentage of patients.

CDT, cold detection threshold; CPT, cold pain threshold; DMA, dynamic mechanical allodynia; HPT, heat pain threshold; MDT, mechanical detection threshold; MPS, mechanical pain sensitivity; MPT, mechanical pain threshold; VDT, vibration detection threshold; WDT, warm detection threshold; WUR, wind-up ratio.

### 3.4. Cold detection threshold and mechanical pain sensitivity correlated for pain intensity and quality of life

There was a negative correlation for CDT with pain severity and functional impairment (Table 3). Loss of function in CDT was associated with higher scores in AWP and severe functional impairment in both men and women. There was a positive correlation for MPS with pain severity, symptoms, and rFIQ in female patients, but not in male patients (Table 4). There was no correlation between other QST parameters with pain intensity and scores of rFIQ.

**Table 3:** Correction between cold detection threshold and clinical presentations.

Scores	AWP	Function	Symptoms	rFIQ
CDT				
Male (n = 38)				
Ƭ_b_ correction coefficient	−0.350	−0.239	−0.167	−0.203
*P*	0.004**	0.041*	0.153	0.079
Female (n = 38)				
T_b_ correction coefficient	−0.242	−0.254	−0.152	−0.194
*P*	0.048*	0.029*	0.189	0.089
Male + female (n = 76)				
T_b_ correction coefficient	−0.209	−0.174	−0.090	−0.110
*P*	0.013*	0.031*	0.263	0.165

Linear corrections were assessed by the Kendall tau-b correction coefficient.

*P < 0.05 and **P < 0.01.

AWP, average weekly pain score; function and symptoms are domains in the rFIQ; CDT, cold detection threshold; rFIQ, revised fibromyalgia impact questionnaire.

**Table 4:** Correction between mechanical pain sensitivity and clinical presentations.

Scores	AWP	Function	Symptoms	rFIQ
MPS				
Male (n = 38)				
Ƭ_b_ correction coefficient	0.061	0.016	0.035	0.007
*P*	0.616	0.890	0.762	0.950
Female (n = 38)				
T_b_ correction coefficient	0.242	0.216	0.314	0.272
*P*	0.048*	0.063	0.007**	0.017*
Male + female (n = 76)				
T_b_ correction coefficient	0.173	0.099	0.172	0.139
*P*	0.040*	0.216	0.032*	0.079

Linear corrections were assessed by the Kendall tau-b correction coefficient.

*P < 0.05 and **P < 0.01.

AWP, average weekly pain score; MPS, mechanical pain sensitivity; function and symptoms are domains in the rFIQ; rFIQ, revised fibromyalgia impact questionnaire.

## 4. Discussion

In this study, we analysed the clinical features and sensory profiles of 38 male and 38 age-matched female patients. Our findings were:

(1) There was no statistical difference in BMI, WPI, SSS, and incidence of depression.
(2) Women had significantly higher scores in pain, sleep interference, and rFIQ, indicating severe symptoms and compromise in quality of life.
(3) Loss of function of small fibres identified by an increased CDT or WDT was seen in 34% of male and 26% of female patients. Cold detection threshold negatively correlated with pain severity and functional impairment, suggesting an important role of small fibre pathology.
(4) Central sensitization was found in two-thirds of male and female patients. Mechanical pain sensitivity correlated with pain intensity and quality of life in female, but not in male patients. Our data confirmed the major role of central sensitization in women.

### 4.1. Gender difference in pain severity and quality of life

Our study has shown that female patients reported severe pain and lower quality of life in comparison with male patients. Earlier studies have found significant differences in the clinical characteristics and the spectrum of fibromyalgia in male and female patients.^6,50^ Gender influence on pain, symptomatology, and psychological comorbidities has been established by recent research.^2,7,14,20,38^ We have confirmed that women had significantly higher scores in overall body pain.^7,20^ The fibromyalgia impact questionnaire (FIQ) has good psychometric properties and showed good correction with comparable domains in the quality of life.^4^ The differences in FIQ between men and women are inconsistent. For example, Aparicio and coauthors have found higher scores in impact, functional impairment, and total FIQ in men.^2^ The FIQ score was not significantly different in Spanish men and women with fibromyalgia,^38^ whereas other authors showed higher FIQ values in women than men.^14,20^ A cross-cultural comparison has found that Spanish and American men experienced similar levels of pain severity, but Spanish men reported higher FIQ values.^24^ Male patients with fibromyalgia reported worse quality of life than depressive men.^48^ Several factors might contribute to the discrepancies, including small sample sizes, and different methodologies such as epidemiology surveys and clinical studies. Furthermore, the quality of life in men should include their masculine identity such as vitality and sexual function.^9,14^ Therefore, FIQ might not be the best tool to assess functional impairment and psychological impact in men.

There may be a tendency to overestimate the clinical importance of small differences in scores that reach statistical significance. We have confirmed the 1-point difference in pain severity on the 11-point NRS between the male and female patients.^7,20^ The minimum clinically significant difference in VAS pain scores is 9 mm (95% CI, 6–13 mm).^21^ Therefore, the gender difference in pain severity was statistically significant and likely clinically relevant. We have found an 11point difference in rFIQ between men and women that was in line with previous findings of differences from 7.5 to 13.^2,14,20^ A double-blind, multicentre trial comparing duloxetine with placebo found a treatment difference of 5.53 in FIQ scores (0–80) associated with the significant favourable patient global impression of improvement and quality-of-life measures.^3^ We attempted to speculate that our finding of an11point difference in rFIQ between men and women was clinically important.

### 4.2. Central sensitization and peripheral mechanism

Every single QST abnormality could occur in neuropathic pain syndromes and fibromyalgia but with different frequency. For example, thermal and mechanical hyperalgesia were more frequent in complex regional pain syndrome (CRPS).^28^ By contrast, postherpetic neuralgia (PHN) displayed thermal and tactile deficits without mechanical hyperalgesia.^33^ We have found a similar sensory pattern in fibromyalgia that is hyperalgesia to nociceptive and hypoesthesia to non-nociceptive stimuli. Mechanical hyperalgesia (MPS 37% and DMA 28%) occurred more often than thermal hyperalgesia (CPT 7% and HPT 12%), which is consistent with what has been shown in neuropathic pain syndromes.^28^ Dynamic mechanical allodynia suggests a hyperexcitable state of central nociceptive neurons, a phenomenon that does not occur in normal health controls. Dynamic mechanical allodynia was present in approximately 20% of patients with neuropathic pain syndromes but can be as high as 50% in PHN.^28,33^ We have found DMA in 29% and 26% of male and female patients, respectively. Wind-up ratio is induced by repeated noxious stimuli, and reflects the temporal summation of pain. We have found WUR in 11% and 13% of male and female patients, which is similar to CRPS, but less frequent than PHN and peripheral nerve injury. We have found that the majority of patients with fibromyalgia had evidence of central sensitization with or without peripheral sensory deficit. In keeping with a recent study,^29^ we have confirmed that a gain of function in MPS is the most dominant gain of function phenotype in both male and female patients.

Fibromyalgia is considered as a “central pain” disorder. Kosek et al.^22^ have found a generalised increase in sensitivity to thermal and mechanical stimuli in patients with fibromyalgia. Thermal and mechanical hyperalgesia was confirmed by other groups.^11,19,41^ A positive correlation was found between central sensitization and pain intensity.^19,35^ In addition, we have found a positive correlation between central sensitization and quality of life in women, but not men.

Assessment of thermal detection thresholds (CDT and WDT) has been widely used to investigate the function of small nerve fibres.^45^ The overall incidence of loss of function in small fibres was found in 40% of patients with various neuropathic pain conditions.^28^ We have found impaired small fibre function in a third of male and a quarter of female patients, which was consistent with previous studies.^13,29^ In comparison, approximately 50% of patients with fibromyalgia have reduced density of somatic intraepidermal nerve fibres (IENF) on skin biopsy.^17,32,43^ The discrepancy could be attributed to significantly lower diagnostic efficiency of QST compared with skin biopsy in detecting SFP (small fibre pathology),^12^ or the limited correlation between functional impairment and structural SFP.^13,25^ For example, symptoms of SFP were found in patients with fibromyalgia in the absence of structural SFP.^25,29^ Evidence has shown that the presence of SFP has little impact on somatosensory symptoms and signs of fibromyalgia.^13^ It appears that a reduction in thermal detection is more clinically relevant than the presence of structural SFN.^49^ Our data have revealed a negative correlation between CDT and pain severity. Cold detection threshold was also found to be negatively correlated with functional impairment in both male and female patients. Our data may suggest an important role of small-fibre sensory dysfunction in the pathogenesis of fibromyalgia.

### 4.3. QST profile is influenced by pain biology and psychosocial factors

The interpretation of QST profiles requires the consideration of gender differences in pain biology and psychosocial factors. It is well established that pain experience and reporting are different in men and women due to distinct pain biology and social factors.^44^ We have found a marked gender difference in MDT and to a lesser degree in VDT. Loss of function in MDT was found in 39% and 16% of male and female patients. This discrepancy could be caused by gender-related pain biology or fibromyalgia. Healthy men were found significantly less sensitive to thermal and mechanical detection on the face.^49^ Gender has a prominent effect on sensory responses in the finger indicating by higher mean sensory nerve action amplitude in women than men.^1^ Furthermore, psychological factors such as depression and PTSD alter sensory interpretation.^9,38^ Therefore, gender-related pain biology and psychosocial factors are likely manifested in QST profiles in patients with fibromyalgia.

### 4.4. Limitations of the study

This is a single-centre retrospective study. The scores of pain severity and quality of life were based on medical records. The scores of pain and questionnaires could be altered by patients' medications. Quantitative sensory testing, a psychophysical assessment, is influenced by the patient's ability and willingness to cooperate. Data on pressure pain threshold was not included in this study as it was not consistently tested. We included the data on vibration detection recorded as absent or present, which deviated from the standard protocol.^36^

## 5. Conclusion

The cardinal symptom of fibromyalgia is chronic widespread pain. There were significant gender differences in pain and quality of life as women reported higher pain scores and lower quality of life. Loss of function of CDT and gain of function of MPS are the most relevant to pain severity and functional impairment. Central sensitization, defined as hyperalgesia and allodynia, was detected in approximately two-thirds of male and female patients. Our data confirm the important role of central sensitization, particularly in female patients. One-third of male and a quarter of female patients had a loss of function of small fibres, and the latter was negatively correlated with pain intensity and quality of life. This study reveals a peripheral mechanism in the pathogenesis of fibromyalgia.

## Disclosures

The authors have no conflict of interest to declare.
